# Current Status of Bioinks for Micro-Extrusion-Based 3D Bioprinting

**DOI:** 10.3390/molecules21060685

**Published:** 2016-05-25

**Authors:** Amit Panwar, Lay Poh Tan

**Affiliations:** School of Materials Science and Engineering, Nanyang Technological University, Block N4.1, 50 Nanyang Avenue, Singapore 639798, Singapore; amit005@e.ntu.edu.sg

**Keywords:** 3D printing, bioprinting, biopolymers, bioinks, printability, resolution

## Abstract

Recent developments in 3D printing technologies and design have been nothing short of spectacular. Parallel to this, development of bioinks has also emerged as an active research area with almost unlimited possibilities. Many bioinks have been developed for various cells types, but bioinks currently used for 3D printing still have challenges and limitations. Bioink development is significant due to two major objectives. The first objective is to provide growth- and function-supportive bioinks to the cells for their proper organization and eventual function and the second objective is to minimize the effect of printing on cell viability, without compromising the resolution shape and stability of the construct. Here, we will address the current status and challenges of bioinks for 3D printing of tissue constructs for *in vitro* and *in vivo* applications.

## 1. Introduction

Even a few decades back, 3D printing of tissues and organs was suggested to cater to the needs of patients who are on organ donation waiting lists [1]. Although many tissue systems have been printed and tested for implantation in animals, success in clinical trials has not been achieved. Apart from just focusing on these constructs for transplantation, 3D printed tissues would be ideal for the development of *in vitro* models for studying diseases, drug screening [2,3] *etc.* 3D printing offers degrees of freedom to imitate natural tissue system due to its potential to control the spatial arrangement of cells. It also provides a platform to make a heterogeneous construct of different cells and bioinks [4]. The first generation of 3D printing was used to construct biomaterial scaffolds which were then seeded with cells to make tissue constructs. Seeding of the scaffolds leads to non-uniform distribution of cells within the scaffold [5,6]. For homogenous distribution of cells in the construct/scaffold as well as to make heterogeneous platforms with multiple cell types with a restricted cell niche, cell-laden biomaterial constructs were developed [7,8,9,10]. Therefore, bioinks which could encapsulate cells to support such construction became an important field for tissue development.

Many printing technologies like light-mediated stereolithography (SLA) [11,12], selective laser sintering (SLS) of polymeric and metallic powders [13], fused deposition modelling (FDM) of synthetic thermoplastics [14,15], inkjet printing [16] and direct extrusion have been employed for scaffold printing [17]. In case of SLS, SLA and FDM, the processes involve high temperature, powder beds, solvent baths and high energy radiations which make them unsuitable for bioprinting of cell laden constructs. Inkjet and extrusion printing are the two major printing technologies which can print cell-laden constructs under physiological conditions. Inkjet printing has been widely used for 3D printing of cell-laden constructs due to its ability to provide good cell viability in comparison to micro-extrusion printing, but bioprinting of viscous bioinks is relatively challenging. This led researchers to employ micro-extrusion printing to print viscous bioinks. Micro-extrusion printing provides a platform to print cell-laden constructs efficiently and in a controllable manner under physiological conditions [18].

In micro-extrusion printing, desired biomaterial structures can be built by dispensing biomaterials through nozzles or needles connected to cartridges loaded with ink. Multiple cartridges can be loaded in the printer to print heterogeneous structures. For bioprinting of cell laden constructs, cells are blended with bioink. Bioink is a material which is used to encapsulate cells to provide a supportive extracellular matrix (ECM) environment and safeguard cells from the stresses a cell has to undergo during printing. Before bioprinting, printing speed, dispensing pressure and movement distance need to be determined for an efficient printing. All the printing parameters depend majorly on the cell line and bioink properties. Printability for a bioink can be determined by the ease with which it could be printed with good resolution and maintenance of its structure after printing. Printability of a bioink can usually be measured by the shape fidelity, resolution, biocompatibility and cell supportive ability [18]. Many researchers have printed cell-laden structures through extrusion printing and have also developed heterogeneous tissue constructs with multiple cell lineages (summarized in Table 1 and Table 2). Although homogenous cell distribution within the construct has been achieved, cell viability gets compromised due to stress conditions that a cell experiences during printing. Direct cell printing will compromise the cell viability but printing of cells by blending with hydrogel has been shown to improve the cell viability.

Despite the many bioinks that have been formulated, development of bioinks still needs significant research to achieve better printability and cell function. One of the reasons is to cater for the differences in cell phenotypes. Different cell lines may have a similar set of genes but a small variation in the gene expression can result in significant differences in phenotype [19]. The phenotype of a cell can broadly be defined by its membrane composition, environment and cytoskeleton [20]. Different cell types have significant differences in their mechanical integrity or phenotypes and develop extracellular matrices of different composition. Also, the microenvironment of the cell makes a significant difference in cell mechanical integrity which varies with location within an organism and among different organisms. This implies that bioinks should ideally be developed specifically for different cell types. Furthermore, for a particular bioink composition and cell type, the range of printing parameters needs to be redefined to get a construct of good resolution and shape.

In an ideal situation, the viscosity of a bioink, supportive for a particular cell type should be somewhat similar or close to a bioink viscosity that is suitable for printability, but both cell supportive and printable viscosities are different. This implies that either the cell has to undergo a stress while printing or printability is compromised. Many researchers have tried to improve the printability of bioinks with cell supportive consistency by varying the printing parameters or by changing the printing strategies [21]. In short, to achieve high cell viability and printability, both printing conditions and bioink consistency have to be optimized.

For bioprinting, Newtonian and non-Newtonian bioinks have been employed and have been optimized for better printable viscosity. Printable viscosity for non-Newtonian bioinks can be determined by the amount of strain rate during bioprinting that further depends on the concentration of bioink and its molecular weight. Moreover, the nozzle shape, size and temperature (for temperature-sensitive bioinks) also contribute to the strain rate during bioprinting. For shear thinning bioinks, viscosity decreases with the increase in strain rate and this helps in safeguarding cells as well as improves the resolution. Shear thinning limits the entanglement of chains due to sliding of chains over each other which allows smooth extrusion of viscous bioink through the nozzle. Due to this, many researchers have tried to enhance the shear thinning properties of bioinks [22].

**Figure 1:**
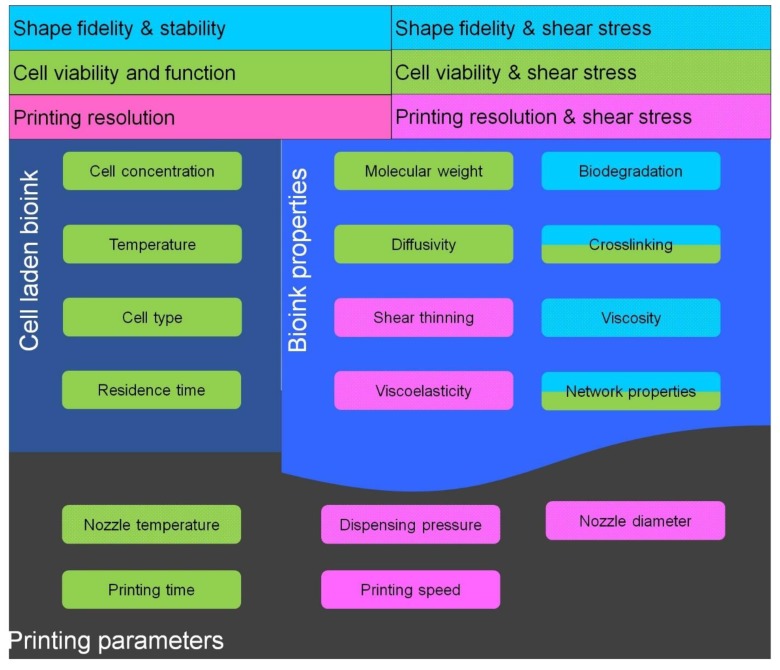
Relationship between bioink consistency, cell laden bioink and printing parameters; factors which control cell viability. Green, factors which control resolution; pink, factors which control shape fidelity and stability; blue, factors which controls shear stress; dot pattern, factors which regulate cell viability along with resolution; blue-green. (Threshold for nozzle and chamber temperature is 37 °C).

Printing conditions majorly include the dispensing pressure, printing speed, nozzle diameter, nozzle temperature and chamber temperature; whereas bioink consistency majorly includes its molecular weight, type and extent of crosslinking, shear thinning properties, gelation point, viscosity, viscoelasticity and hydrophilicity. The relationships between printing parameters, bioink consistency and cell laden bioink are shown in Figure 1.

**Figure 2:**
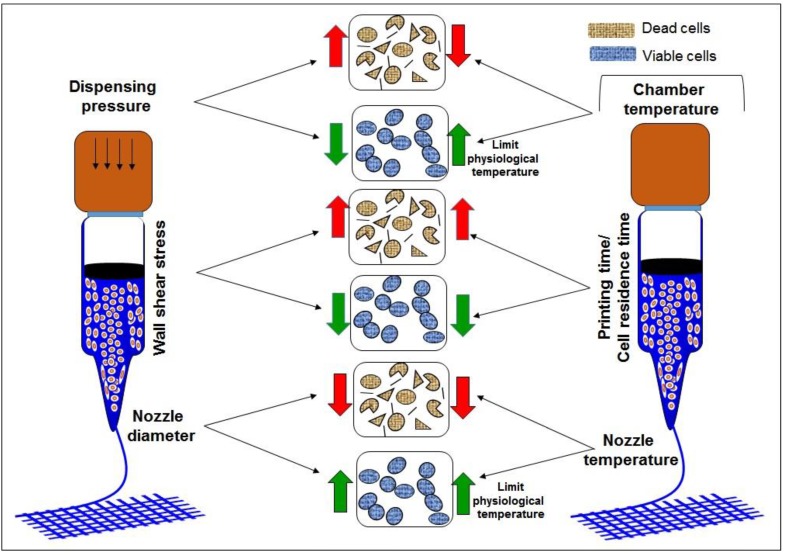
Effect of various printing parameters on cell viability. dispensing pressure increase would increase the shear stress which will decrease the cell viability; wall shear stress is due to walls of nozzle and cartridge which decreases the cell viability and depends upon dispensing pressure and nozzle diameter and bioink concentration; nozzle diameter decrease would decrease the cell viability ; chamber temperature increase will increase the cell viability up to threshold 37 °C and viability decrease with decrease in temperature for thermal sensitive bioinks; printing time increase would decrease cell viability due longer exposure of cells to printing environment; nozzle temperature decrease would decrease cell viability for thermal sensitive bioinks.

These parameters play an important role in getting a good resolution construct as well as good cell viability [41]. Different bioinks respond differently to shear force, affecting especially the bioink present near to syringe and needle walls. Dispensing pressure, bioink consistency and nozzle diameter usually determine the shear force experienced by the cells encapsulated in the bioink and affects the cell viability as shown in Figure 2. With an increase in nozzle diameter, shear stress will decrease due to which cell viability increases but the resolution gets compromised. Another major issue is dependence of cell viability on concentration of bioinks. For, majority of bioinks, cell viability decreases with an increase in concentration, especially in case of gelatin and alginate. Higher concentrations of bioink prevent cell migration and diffusion due to an entangled network, which leads to lower cell viability. Through this review, we report on the printability of current bioinks and their cell supportive behavior under a set of printing parameters. We will discuss both nature derived and synthetic bioinks.

## 2. Nature-Derived Bioinks

Nature-derived materials play a vital role to fabricate cell supportive microenvironments for bioprinted cells. In this section, bioinks derived from natural sources are discussed and their bioprinting potential explored. Both animal and plant derived bioinks have been utilized for bioprinting applications. In comparison to plant-derived bioinks, animal-derived bioinks facilitate better cell growth and function. Shear thinning is one of the properties which is common among all bioinks, due to non-covalent interactions between the molecules. Generally, nature-derived bioinks have greater potential to support cell viability and growth than synthetic bioinks, however, synthetic bioinks provide an ease of tailoring their properties for efficient printing, whereas modification of nature-derived bioinks is difficult and limited. Herein, we will discuss some of the most reported nature-derived bioinks.

### 2.1. Alginate-Based Bioinks

Alginate is an anionic polysaccharide of mannuronic acid and guluronic acid obtained from seaweeds and algae. It forms a tough hydrogel upon addition of calcium ions due to sodium-calcium ion exchange reactions. It has been used as a promising hydrogel for tissue engineering due to its inert nature. Although it lacks cell binding domains, blending or modification with molecules having cell binding domains improves its cell adhesion.

#### Printability and Challenges

Alginate hydrogels have shear thinning properties and therefore, their viscosity is dependent on the strain rate. Previous reports suggest that the viscosity of alginate is concentration dependent, whereby a fivefold increase in concentration can increase the viscosity by up to 100-fold. The nature of the alginate hydrogel can vary from forming a soft hydrogel with low viscosity below 5% *w*/*v* concentration to viscous hydrogels above this concentration [58].

Generally, lower concentrations of alginate are recommended for high cell viability. However, at lower concentration, achieving good resolution for printing applications is challenging. Many attempts to optimize the resolution of alginate bioinks have been reported, including optimization of alginate concentration, blending with high molecular weight polymers and tuning of printing parameters. Like the viscosity, the resolution of alginate bioinks is also dependent on concentration. Studies have reported that at 0.5 *w*/*v* concentrations of alginate, a decrease in nozzle diameter would decrease the drop volume by almost 3-fold thus increasing the resolution. However, at a higher alginate concentration of around 1.5% *w*/*v*, decreasing the nozzle size would instead increase the drop volume by 3-fold and thus negatively affect the resolution. The decrease in viscosity due to shear thinning at higher concentrations has been indicated to be responsible for the increase in drop volume. Although in the above reported study no constructs could be fabricated due to the low viscosity of alginate, the effect of nozzle diameter and concentration on resolution were successfully established [71].

Alternatively, blending of alginate with high molecular weight molecules could improve the resolution of alginate printing. Blending with nano-fibrillated cellulose has enhanced the resolution from 1000 µm to 400–600 µm, when printed with a 300 µm diameter nozzle [38]. The improvement in resolution by this blending is attributed to the shear thinning property and high viscosity of bioink due to the presence of nanocellulose. Other attempts involve optimization of printing parameters which are of utmost importance for better resolution. Optimization of printing parameters like nozzle size, printing speed and dispensing pressure can help in improving the resolution. The printing speed and dispensing pressure has been optimized by Dhert and coworkers. An increase in dispensing pressure from 0.15 to 0.25 MPa decreased the resolution by increasing the strut size from 400 to 1600 µm at 100 mm/min printing speed, whereas an increase in printing speed from 100 to 300 mm/min increased the resolution by decreasing strut size from 1600 µm to 1000 µm at 0.25 MPa dispensing pressure [10].

A major disadvantage of alginate bioinks is their formation of soft gels at lower concentrations. To overcome this issue, various strategies have been implemented to improve shape fidelity, including crosslinking, prefabricated scaffolds and sacrificial scaffolds [52]. One strategy is crosslinking by calcium ions, which has been widely employed by researchers [38,51,72]. For crosslinking, printed constructs need to be exposed to CaCl_2_ solution. This exposure leads to the exchange of sodium ions with calcium ions whereby the divalent calcium ions will form a crosslink between two carboxyl groups of the same or different polymer chains. However, such crosslinking cannot provide sufficient mechanical strength to hold its 3D printed structure. Another strategy involves the use of concomitantly printed supportive scaffolds along with crosslinking to improve shape fidelity. A study reported by Cho and coworkers employed concomitantly printed PCL scaffolds for bioprinting of cell laden alginate bioinks [51]. Apart from these supportive scaffolds, sacrificial scaffolds were also reported to enhance the shape fidelity and to print complex structures [49,52]. In sacrificial scaffolds-based printing, a scaffold with a mirror image of the desired structure is printed simultaneously with a cell laden bioink, and is then removed after gelation of the cell laden bioink without affecting the shape of the printed construct. A successful fabrication of ear-shaped constructs using PEG sacrificial scaffolds for the fabrication of cell laden alginate bioink constructs supported with PCL scaffolds has also been accomplished [52]. In the study, a PCL scaffold with alginate bioink was printed within a PEG scaffold to support the cell laden alginate bioink. After PCL layer printing, cell laden alginate bioink was printed in the spaces between PCL struts and further printed cell laden alginate bioink was crosslinked with CaCl_2_. Printing of all the three components was coordinated in a way to achieve an ear shaped construct after removal of the sacrificial layer.

As the amount of stress generated inside a bioink depends on the properties of the bioink, alginate bioinks provide some form of shielding to the cells from the printing process stress. A study has reported 90%–94% viability for printing of chondrocytes and osteoblasts when printed with 4% *w*/*v* alginate bioinks in a PCL scaffold [50]. Although alginate bioinks safeguard cells from printing stress, they lack cell binding domains to support cell growth and function. Attempts have been made to improve the cell binding ability by modification with peptides and blending biopolymers with cell binding domains. A study that reported bioprinting of human adipose derived stem cells using RGD-modified alginate bioink showed improved cell attachment and integrin expression [58]. Other studies also highlighted that RGD-modified alginate improved cell attachment, growth and function in comparison to native alginate [72]. Alternatively, the blending of gelatin with alginate has improved the cell supportive ability of alginate bioinks [25]. Gelatin has cell binding ability due to presence of RGD domains and therefore, an increase in the proportion of gelatin in alginate bioinks improved the cell viability and function.

Even though alginate has been one of the most studied bioinks, there remain many challenges. Firstly, it has been difficult to produce high shape fidelity with alginate hydrogels. The crosslinking of alginate is not rapid enough to maintain a high shape retention. To improve the resolution, blending with high molecular weight polymers has been attempted but cell viability is compromised. Secondly, as alginate forms a relatively soft gel, even after crosslinking, it is challenging to print multilayered structures which can recapitulate complex tissues structures. Lastly, as mentioned above, the absence of cell binding domains in alginate makes this material not supportive of cell attachment and proliferation.

### 2.2. Gelatin-Based Bioinks

Gelatin is a hydrolyzed collagen obtained from various sources like pork or calf skin and fishes. Depending on the type of hydrolysis it is categorized into two types: gelatin A and gelatin B. Gelatin A is obtained through acid-based hydrolysis of collagen and gelatin B is produced by base-based hydrolysis of collagen. Gelatin displays the capability to form physically crosslinked hydrogels at low temperature and has suitable biological properties that can support cell growth and function. All these properties make it suitable for substitution in tissue engineering applications.

#### Printability and Cell Support

Gelatin and its derivatives have been widely employed for 3D bioprinting due to their biocompatibility, biodegradability and cell binding RGD domains [73]. Pure gelatin is soluble in water and forms a thermosensitive hydrogel that undergoes a sol-gel transition below the critical solution temperature (25–35 °C). At physiological temperature, pure gelatin forms a low viscosity solution making it difficult to use for bioprinting applications. One approach to enhance the viscosity is through blending of gelatin with other viscous polymers. A study has reported blending of gelatin methacrylamide (GELMA) with gellan gum increased the viscosity by more than one order of magnitude due to interactions between the negatively charged deprotonated glucuronic acid of gellan gum and the positively charged lysines of gelatin methacrylamide. Further increases in viscosity by two orders could be achieved by addition of monovalent cations [28]. Other attempts include blending of gelatin with alginate [34], hyaluronic acid [29,63], fibrin [74] and silk [75] to enhance the viscosity for bioprinting. To increase the viscosity further, a study has reported partial crosslinking of blends of polyethylene glycol polymer derivatives with gelatin prior to printing [33].

**Figure 3:**
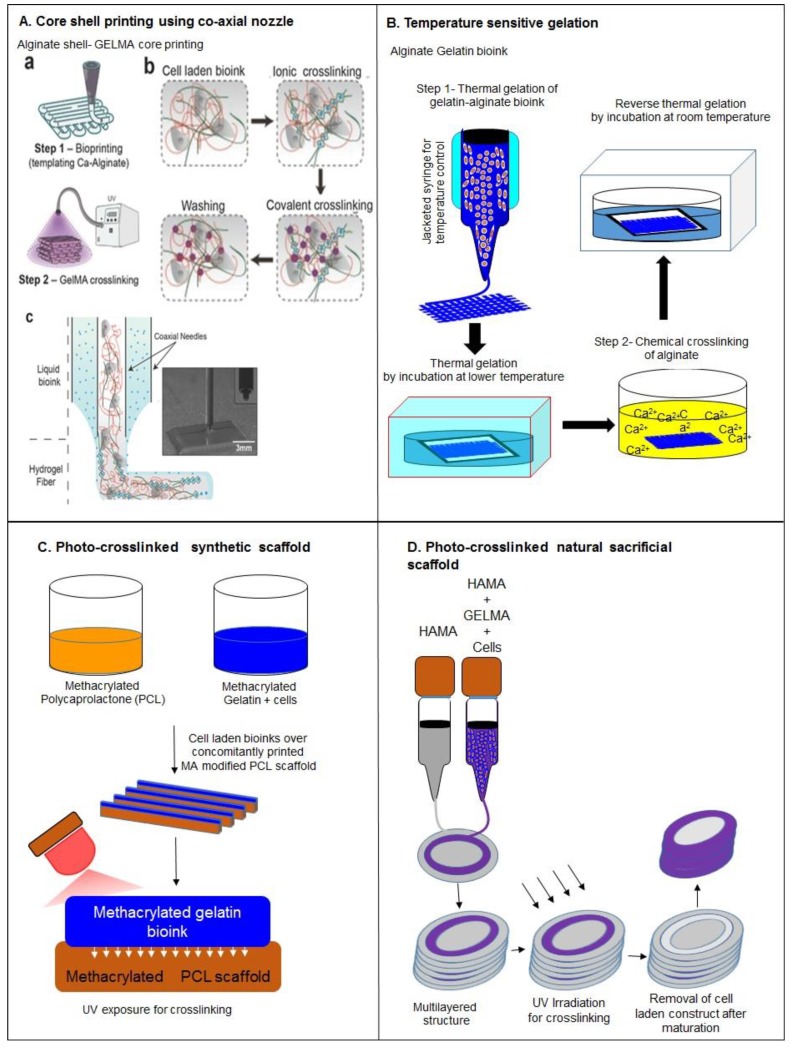
(**A**) Printing of GELMA-alginate blend using coaxial nozzle with simultaneous crosslinking. Reproduced with permission form [34]. Copyright 2016, John Wiley and Sons; (**B**) Temperature sensitive gelation of gelatin alginate bioink; (**C**) Photo-crosslinked PCL scaffold with gelatin methacrylamide; (**D**) Photo-crosslinked natural HAMA scaffold for ring structure.

Another disadvantage of gelatin bioinks is their poor bioprinting resolution. For better bioprinting resolution blending with other polymers is again an effective strategy. In the same study as above that involved gelatin methacrylamide (GELMA) with PEG, derivatives, blending and partial crosslinking of the resolution was improved, as shown by the reduction of strut size from 1100–1300 µm to 350–450 µm, when bioprinted with a 200 µm diameter nozzle [33]. An extensive study on the use of methacrylated gelatin bioink has been reported by Khademhosseini and coworkers wherein they could reduce the strut size to as low as 150–200 µm strut diameter for gelatin methacrylamide-alginate bioink printed using a co-axial nozzle. In the reported study, for crosslinking of alginate, crosslinking solution (CaCl_2_) was dispensed through a co-axial nozzle which could provide simultaneous crosslinking during extrusion as shown in Figure 3A [34]. Other attempts to improve gelatin-based bioprinting involve optimization of printing parameters to achieve better resolution. For instance, a decrease in strut size from 700 to 250 µm can be achieved by increasing printing speed from 100 to 1000 mm/min [46]. Other studies have stated that blending of gelatin with alginate gives better resolution. A strut size of 400–450 µm has been achieved for gelatin when blended with 4% alginate [23,27,42].

Moreover, bioprinting using gelatin bioinks also from suffers poor shape fidelity. For shape fidelity, crosslinking after printing has been reported as an effective strategy. A study has reported two step crosslinking by using thermosensitivity of bioink constituents for better shape fidelity [76]. The two step crosslinking of gelatin alginate bioink using thermosensitivity has been demonstrated to improve the shape fidelity, where, first gelatin was crosslinked physically (during printing) by lowering the temperature and later (after printing) alginate was crosslinked using CaCl_2_ solution shown in Figure 3B. GELMA is another easy and popular way to induce photo-crosslinking after printing in order to maintain shape fidelity. Mechanical integrity of GELMA relies on the number of crosslinking moieties and UV exposure. A study has observed increase in the elastic modulus from 20 to 50 kPa by increasing the UV exposure from 15 s to 60 s [34]. Further, the UV crosslinking of GELMA with a supportive polymer can significantly enhance the shape fidelity of the constructs [76]. In another study, the supportive polymer hyaluronic acid methacrylamide (HAMA) was blended with GELMA and was photo-crosslinked after bioprinting of cell laden constructs [64,77] which could stabilize the shape fidelity of the construct. Apart from photo-crosslinking, enzymatic crosslinking by using transglutaminase has also been established to improve the shape fidelity of printed cell laden constructs [78].

**Figure 4:**
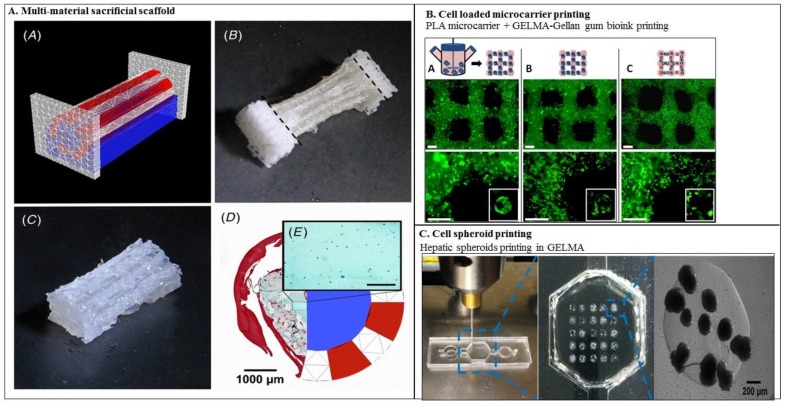
(**A**) Design of a fiber reinforced single tube (inner diameter 4 mm, outer diameter 6 mm, length 20 mm) including anchoring stands (white: PCL, red: Gelma-gellan, blue: alginate); (**B**) construct directly after printing, scalpel cuts for removing stands represented by dashed lines; (**C**) tube after removing PCL stands; (**D**) cross-section of printed tube, right: design, left: after infusion with gelatin containing MSCs (safranin-*O* staining, red: Gelma-gellan tube wall; green: Gelatin-MSC mixture). Reinforcing PCL fibers that were present in the tube wall were dissolved during the embedding process; (**E**) magnification from picture D of gelatin hydrogel containing MSCs (blue dots) (scale bar represents 200 µm). Reproduced with permission from [65]. Copyright 2016, IOP Publishing; (**B**) Micro-carrier printing in GELMA bioink. Reproduced with permission from [35]. Copyright 2016, IOP Publishing.; (**C**) HepG2 spheroid printing using GELMA bioink for perfusion bioreactor. Reproduced with permission from [36]. Copyright 2016, IOP Publishing.

An additional approach to improve the shape fidelity of gelatin-based bioinks involves an external support for the gelatin bioink and can be classified into three categories: (a) bioprinting in prefabricated scaffolds; (b) micro-carrier printing; (c) spheroid printing. In the first approach, concomitantly printed supportive scaffolds of natural or synthetic polymers are employed for bioprinting of bioink laden with cells. A study has reported a methacrylate functionalized PCL scaffold to support the cell laden GELMA bioprinted construct where the photo-crosslinking between methacrylate modified PCL and GELMA helps in improving the shape fidelity and mechanical strength of the printed construct, shown in Figure 3C [56]. Sacrificial scaffolds derived from natural as well as synthetic polymers have been also employed to improve shape fidelity. A study reported a HAMA sacrificial scaffold for the bioprinting of ring-shaped cell laden GELMA-HAMA structures where the HAMA scaffolds were removed after fabrication to provide a tubular structure shown in Figure 3D. Sacrificial scaffolds of poly-PCL and alginate have also been utilized to print cell laden gelatin bioink as shown in Figure 4A [65]. The second approach to provide an external support for gelatin bioprinting involves bioprinting of cell laden microcarriers. Cell laden polyvinyl acetate (PVA) microcarriers were bioprinted using GELMA-gellan bioink where the microcarrier printing could mechanically strengthen the bioprinted cell laden strands to retain their shape after extrusion, as shown in Figure 4B [35]. The third approach involves spheroid fabrication and then bioprinting. Spheroids are cell aggregates which either form due to cells’ self-ability or are induced artificially to form spheroids. In a study reported by Khademhosseini and coworkers HepG2 cell spheroids produced through micropatterning were bioprinted using GELMA bioink, as shown in Figure 4C [36].

Although gelatin provides a cell supportive environment to bioinks, its stress shielding properties to protect cells from bioprinting stress are low. In the case of gelatin methacrylamide, a study has reported 70% viability of human neonatal dermal fibroblasts (HNDFs) [53] just after printing, demonstrating lower stress shielding to the cells during bioprinting. One efficient strategy involves optimization of nozzle temperature during bioprinting to improve the cell viability. The study has reported ~90% survival of HeLa cells at 25 °C nozzle temperature, whereas̴ ~50% survival has been reported at 10 °C. A decline in the temperature to 10 °C increased the viscosity of gelatin which resulted in lower cell viability [24]. Another cause for lower cell viability involves UV crosslinking of methacrylated gelatin. For instance, an increase in UV exposure from 15 s to 60 s decreased the viability of human umbilical vein endothelial cells (HUVEC) cells from 70% to 40% on the 3rd day [34] where the decrease in cell viability was attributed to the increase in crosslinking as well as the effect of UV radiation.

Gelatin and its derivatives are some of the nature-derived bioinks that have gained significant attention. Generally, they have good biocompatibility, are cell supportive, biodegradable and easily modified for optimization of printability. The bioprinting of methacrylated gelatin seems to be gaining traction in the recent years due to the above factors and might be a promising bioink if cell viability could be improved. Further work to find an alternative to the acrylate group such that cell viability is enhanced could also be a significant research direction.

### 2.3. Collagen-Based Bioinks

Collagen is the most abundant protein of animal ECM which is made up of three polypeptide chains that forms a triple helix structure and consists of many RGD domains which provide binding support to the cells [66]. There are about 28 different types of collagen proteins that are present in the human body, but mostly type I collagen has been used for tissue engineering applications because of its abundant availability. Moreover, type I collagen undergoes physical crosslinking when incubated under physiological conditions, thereby displaying a suitable property for bioprinting applications.

#### Printability and Challenges

Collagen is derived from natural ECM, which makes it a promising biomaterial for bioink development. However, pure collagen hydrogel bioprinting is difficult due to its low viscosity. To improve the viscosity many attempts have been made and blending with other polymers has been the most studied. Blending with agarose [79], chitosan [80], fibrin [81] and hyaluronic acid [82] has been reported to improve the viscosity of collagen bioinks and the blends are printable as well.

Apart from increasing the viscosity, blending with polymers has also been reported to improve the shape fidelity. In a study of collagen-agarose blend, the printed cylinder height was able to increase from 2 mm to 4 mm after blending with 3% agarose. The construct was able to maintain its shape for 24 h. [79]. Alternatively, physical crosslinking of collagen has also been attempted to enhance shape fidelity. Physical crosslinking of collagen was established by two methods. One is by incubation under physiological conditions after printing of each layer, whereas the other involves neutralization of acidic collagen to form a solid gel by exposing it to sodium bicarbonate [83]. Other attempts include use of concomitantly printed synthetic scaffolds with voids for better shape fidelity. In a study reported by Cho and coworkers, scaffold of PCL was used for bioprinting of collagen bioink laden with cells [82].

Moreover, collagen bioinks have the potential to protect cells during printing. Studies reported 86% viability of collagen I laden bovine aortic endothelial cells (BAEC) after bioprinting with a 25 gauge nozzle, but a decrease in nozzle size from 25 to 33 gauge decreased the cell viability to 46% [84]. Along with safeguarding of cells, collagen bioinks support cell function and viability in printed cell laden constructs. Studies have reported better cell viability and migration of osteoblasts in bioprinted cell laden constructs of collagen I bioink, demonstrating the cell supportive ability of collagen bioink. Although the blending of collagen bioinks with other biopolymers improves the printability and viscosity, it compromises the cell growth in the printed construct. Studies have reported blending of agarose with collagen would decrease the cell growth of mesenchymal stromal cells by 3-fold in bioprinted constructs when compared with pure collagen [79].

Although the printability of collagen bioink has been improved, weak mechanical strength and fast hydrolysis still represent major challenges for its use in bioprinting. Nevertheless, collagen-based bioinks display enormous potential to support cell growth and function and therefore, there is a wide scope for development of collagen-based bioinks for bioprinting of soft and hard tissues.

### 2.4. Fibrin-Based Bioinks

Fibrin is a protein formed by mixing of fibrinogen and thrombin and it can easily be crosslinked by incubation of fibrinogen, calcium ions and thrombin. It has often been used in tissue engineering applications due to its cell adherence properties. For 3D bioprinting, its properties can easily be tailored by varying the proportion of its components, which makes it a suitable bioink for 3D bioprinting.

#### Printability and Challenges

Pure fibrinogen forms a hydrogel of low viscosity and therefore, it has been printed through inkjet bioprinting methods. For extrusion bioprinting, it has been used as an additive with other biopolymers due to its excellent bioactivity and mechanical strength after crosslinking. The blending of fibrin with gelatin, alginate and collagen has been demonstrated to provide a bioactive cue and mechanical strength to the bioprinted constructs for better shape fidelity. The mechanical properties of fibrinogen composites have also been tuned, such as increase in the compressive modulus of gelatin fibrinogen constructs from 1.85 N to 2.59 N and elastic modulus from 0.000531 to 0.0259 N/mm^2^ by increasing fibrinogen proportion from one-third to half, after crosslinking [74].

Fibrin gels are fibrinogen crosslinked by incubating it with thrombin at room temperature. Thrombin protease cleaves the fibrinogen from two points leaving symmetrical structures, which then aggregate non-covalently. The major disadvantage of using fibrin is its fast and irreversible gelation at body temperature, which makes its bioprinting difficult [85]. To overcome this, thrombin and fibrinogen blends can either be printed at low temperature together to prevent early crosslinking or separate thrombin deposition can be carried out over a construct for crosslinking after bioprinting of the 3D construct [24].

During application, the biodegradability of material should match the rate of natural synthesis of native ECM by the cells, but, fibrin undergoes rapid degradation which impairs its ability to form stable structures. However, various attempts to improve the stability have been established, such as blending with other biopolymers like gelatin and alginate. Of the two biopolymers, blending with alginate hydrogels provides improved stability, whereas blending with gelatin results in formation of unstable structures due to the biodegradable nature of gelatin [27].

In terms of cell support and viability, fibrin gel is cell supportive in nature and enables sufficient maintenance of cell growth and function. It has also been shown that the blending of fibrin with gelatin alginate bioinks results in similar cell viability as compared to only gelatin alginate structures [42].

### 2.5. Hyaluronic Acid-Based Bioinks

Hyaluronic acid (HA) is a glycosaminoglycan biopolymer with disaccharide unit repeats of d-glucuronic acid and *N*-acetyl-d-glucosamine. It is a component of the extracellular matrix. Inside tissues, it plays many important roles, including maintenance of structural integrity and hydration of ECM, tissue homeostasis and influence cell growth, migration and differentiation.

#### Printability and Challenges

Hyaluronic acid hydrogel has high viscosity for bioprinting. Due to this high viscosity it has been used as a supportive hydrogel to modify the viscosity of bioinks. The viscosity of HA-supported bioinks is concentration dependent. A study reported an increase in the viscosity of HA gelatin blended bioinks from 10 Pa·s to 1000 Pa·s with an increase in concentration of HA from 2% to 4% *w*/*v* at room temperature [77]. Although HA has high viscosity, cell laden constructs cannot hold the 3D bioprinted structure due to poor mechanical strength. The low mechanical integrity of HA results in poor shape fidelity. Various attempts have been made to improve the shape fidelity including, crosslinking [29] blending [63] and use of synthetic scaffolds for bioprinting. Hyaluronic acid crosslinking has been successfully accomplished by physical means and photochemically. In physical crosslinking, HA bioinks was incubated at room temperature [82], whereas in photochemical crosslinking HA was modified with methacrylate [63]. Methacrylated hyaluronic acid (HAMA) undergoes crosslinking on UV exposure in the presence of a photoinitiator. In addition to crosslinking, blending of HA and its derivatives with photochemical crosslinked polymers further improved the shape fidelity. Studies have reported bioprinting of cell laden constructs of hyaluronic acid methacrylate (HAMA)-GELMA bioink and HA-PEG tetraacrylates, which demonstrate potential of photocrosslinking to improve shape fidelity. Other attempts include use of simultaneously printed synthetic scaffolds, bioprinting of cell laden HA bioinks in PCL scaffolds has been established for better stability and shape of construct [82].

Although hyaluronic acid is an excellent biopolymer in terms of viscosity, the stability of hyaluronic acid bioinks is challenging. It undergoes fast hydrolysis due to its hydrophilicity. Many attempts have been reported to improve stability including semi-interpenetrating hydrogels and blending. Studies reported by Vermonden and coworkers demonstrated improved stability of hyaluronic acid—dextran hydroxyethyl methacrylate-based semi-IPN hydrogels employed for chondrocytes bioprinting. In addition, UV crosslinking of dextran-HEMA improved the stability of the final construct [30]. As already mentioned, blending with synthetic polymer derivatives for shape fidelity *i.e.*, PEG tetraacrylate, provided better stability due to controlled degradation.

Just like many hydrogels, HA bioinks provide shielding effects to cells during printing. Cells printed with HA bioinks and its blends could generally have initial viability as high as 90% or more [2,3]. Hyaluronic bioink not only safeguards cells from printing stress but also improves the cell function and viability after printing. In a specific study, it was reported that HA bioink supported better cell migration of chondrocytes in comparison to collagen I bioink. This is due to the fact that HA is the native ECM of chondrocytes [2].

### 2.6. Decellularized Extracellular Matrix

For tissue engineering and 3D bioprinting, researchers have continuously been trying to mimic the natural extracellular matrix (ECM) by modifying synthetic or natural biopolymers. The development of decellularized bioinks have provided a provision to source a native ECM to cells [59].

#### Printability and Challenges

Decellularized extracellular matrix (dECM) is extracted from the animal sources for tissue engineering applications. As it is derived from different tissues, it is rich in cell growth and differentiation factors which help in supporting growth as well as the function of specific cell lines. In general, dECM has low viscosity but it may vary with tissue source. A study reported high viscosity of 23.6 Pa·s^−1^ (15 °C) for heart-derived decellularized bioink, but a low viscosity of 6.13 and 2.8 Pa·s^−1^ for adipose and cartilage-derived decellularized ECM, respectively [59]. Due to the general low viscosity of dECM they display low shape fidelity and various attempts have been made to improve this property, including crosslinking and bioprinting in concomitantly printed synthetic scaffolds. One approach to improve the shape fidelity of dECM bioinks is physical crosslinking of dECM. Studies reported physical crosslinking (gelation) of dECM by incubating the physical construct in humidified incubator for 30 min which resulted in an abrupt change in modulus when temperature increased from 4 °C to 37 °C [59]. Mechanical integrity is important to maintain shape fidelity of the final construct. If the crosslinking could not provide sufficient mechanical integrity to hold the cell laden 3D bioprinted structure, an external support can be provided to improve the shape fidelity. The external support can involve the use of co-printed synthetic scaffolds for 3D bioprinting of cell laden constructs. This has been successfully demonstrated by bioprinting of adipose-derived mesenchymal stem cells laden adipose-derived dECM in concomitantly printed PCL scaffolds [60]. Another disadvantage of using dECM bioinks is the poor bioprinting resolution due to low viscosity. A study reported, strut size of around 1100 µm when printed with a nozzle with 250 µm diameter [59]. As only few studies on bioprinting using dECM have been reported, and optimization of resolution needs to be established.

Although dECMs have many challenges in bioprinting, they have been employed for bioprinting due to their cell supportive ability. Greater than 90% cell viability on the 14th day has been reported for bioprinting of human adipose-derived stem cells using decellularized bioinks, which is significantly high in comparison to other bioinks [60]. For a shorter culture time of 24 h, >95% cell viability has been reported [59].

Overall, there are some major challenges with dECMs for bioprinting of cell laden construct that are to be tackled before they can be exploited for extensive application in tissue bioprinting. Firstly, the shape fidelity is low due to the weak mechanical integrity of dECM. Secondly, biodegradation of decellularized bioinks is rapid, and could not match the rate of new ECM generation [67]. Lastly, as mentioned, the resolution is poor due to the low dECM viscosity.

### 2.7. Silk Based Bioinks

Silk is a high molecular weight protein produced by silk worms and spiders. Silk is considered for biomaterial applications due to its non-toxic nature, slow degradation and low immunogenicity. It has shear thinning properties, high viscosity and a β-sheet structure formed during gelation, which assists in the development of efficient bioinks for bioprinting. In the case of 3D bioprinting, silk and its recombinant versions has been used as bioinks [57].

#### Printability and Challenges

Silk has been used as a biomaterial for the fabrication of clinical devices, biosensors and implants. The major advantages of silk are shear thinning, high viscosity, non-toxicity, slow degradation, low immunogenicity and good mechanical properties, which makes it seem to be an ideal biomaterial to be employed for 3D printing. The major disadvantage of pure silk for bioprinting is that the high viscosity results in nozzle clogging during bioprinting [86]. Due to shear forces inside nozzle during printing, the polypeptide chains form β crystallites which clog the nozzle. One efficient strategy to prevent nozzle clogging involves recombinant silk. A study by Scheibel and coworkers has reported bioprinting of cell laden constructs using recombinant silk [86]. Recombinant silk eADF4 was developed from 16 repeats of module C (consensus sequence) present in ADF4 protein of *Araneus diadematus* silk [68]. eADF4 has a lower viscosity in comparison to native silk and hence could avoid clogging during bioprinting. Another strategy involves blending of silk with other polymers. A study reported blending of 15% silk with 10% gelatin to reduce the viscosity of silk which subsequently improved its printability [86]. The viscosity of silk fibroin gelatin bioink becomes temperature dependent, whereby the viscosity is lower just like a sol at 37 °C, and a decrease in temperature from 37 °C to 28 °C would increase the viscosity by 6-fold. Due to this, silk-gelatin was bioprinted at 28 °C [75].

Crosslinking has also been attempted by various groups to improve the shape fidelity of printed silk constructs. In the same study by Scheibel’s group, recombinant silk bioink was incubated overnight before printing at 95% humidity to improve the shape fidelity. Incubation at 95% humidity induces physical crosslinking due to formation of β-sheet structures from random helices which optimizes the viscosity [57]. Another study has reported sonication-based and enzymatic crosslinking. In sonication-based crosslinking, sonication induces β-structures by altering hydrophobic hydration and self-assembly of silk peptides, whereas in enzymatic crosslinking tyrosinase oxidizes the protein residues into *o*-quinones, which either condense with each other or undergo non-enzymatic reactions with nucleophile groups present in the protein [75].

For the fabrication of heterogeneous constructs, good bioprinting resolution with better shape fidelity is required. Strut width of 280–320 µm with 250 µm nozzle diameter with recombinant bioink has been successfully established [57], whereas blending of silk fibroin decreased the resolution to double the nozzle diameter. Blending of silk with gelatin resulted in larger strut size of 626 µm ± 8 µm with a 300 µm nozzle diameter, probably due to the change in viscosity [75].

Furthermore, silk-based bioinks safeguard cells from stress during printing. For silk-fibroin gelatin bioink-based bioprinting, human nasal inferior turbinate tissue-derived mesenchymal stromal cells (hTMSC) have >95% viability on the first day after printing. In case of fibroblast bioprinting with recombinant silk bioink, cell viability was 70.1% ± 7.6% after 48 h of incubation and have relatively 97% viability in comparison to unprinted cells laden in silk-fibroin gelatin hydrogel. For better cell adhesion, recombinant silk was modified with RGD peptide. Fibroblasts cultured over RGD modified silk develop filopodia and have 50%–60% higher cell adhesion in comparison to recombinant silk without RGD [57].

There are many challenges in bioprinting of silk. Firstly, it lacks cell binding domains which limits cell adherence. Secondly, the optimization of silk based bioink has not been established as only a few reports are available. Lastly, lower cell growth and function support. A study has reported a significant decrease in proliferation of fibroblasts with an increase in silk concentration from 5 to 20 mg/mL [69].

### 2.8. Chitosan-Based Bioinks

Chitosan is a polysaccharide with β (1–4) linked d-glucosamine residues with variable number of *N*-acetylglucosamine groups. Properties like non-toxicity, biodegradability, anti-bacterial and anti-fungal activity make chitosan suitable for application in tissue engineering and 3D bioprinting. It shows structural characteristics similar to glucosaminoglycans and hyaluronic acid because of which it has been widely used for cartilage tissue engineering.

#### Printability and Challenges

Chitosan is a high molecular weight biopolymer and forms a viscous hydrogel. The viscosity of chitosan is optimum for bioprinting, as high viscosity is recommended for better printability. The disadvantage of chitosan is poor mechanical integrity, which makes the 3D bioprinted structure fragile and flimsy. One attempt to improve shape fidelity relies on blending of chitosan with other biopolymers. Chitosan has been blended with polymers like agarose, alginate to improve the shape fidelity, but failed to form an intact 3D printed structure. A study reported bioprinting of microfluidic channels using chitosan hydrogel. Although the microfluidic channels were printed successfully, it failed to perform due to disruption of channels [87]. Another study reported blending of chitosan with agarose to print mesenchymal stromal cell laden cylinders, but again it was not successful in holding the final cylindrical construct [79]. Therefore, instead of being the major component, chitosan has been used as the minor component in a blend to support the printability. A study reported improved resolution for collagen-chitosan bioink, wherein collagen chitosan polyelectrolyte hydrogel was developed for bioprinting of neonatal human foreskin fibroblasts [61].

There are many challenges in development of chitosan-based bioinks. Firstly, the mechanical integrity of chitosan is weak, which makes printing 3D structures challenging. Secondly, chitosan undergoes rapid dissociation under physiological conditions. Chitosan hydrogel is stabilized by polyanioinic complexes which undergo dissociation at neutral pH. Dissociation of polyanioinic complexes results in disruption of construct shape and mechanical integrity [64]. Moreover, the presence of these polyanioinic complexes affects cell viability. Lastly, it lacks cell binding domains which limits its application to only a few cell lines.

## 3. Synthetic Bioinks

### 3.1. PEG-Based Bioinks

Polyethylene glycol (PEG) is a hydrophilic polymer with linear and branched structures. It is often used for surface modification, bioconjugation, drug delivery and tissue engineering [70]. PEG and its derivatives are probably the most explored synthetic material for soft tissue engineering applications. PEG is very versatile and its properties could be easily tailored.

#### Printability and Challenges

**Figure 5:**
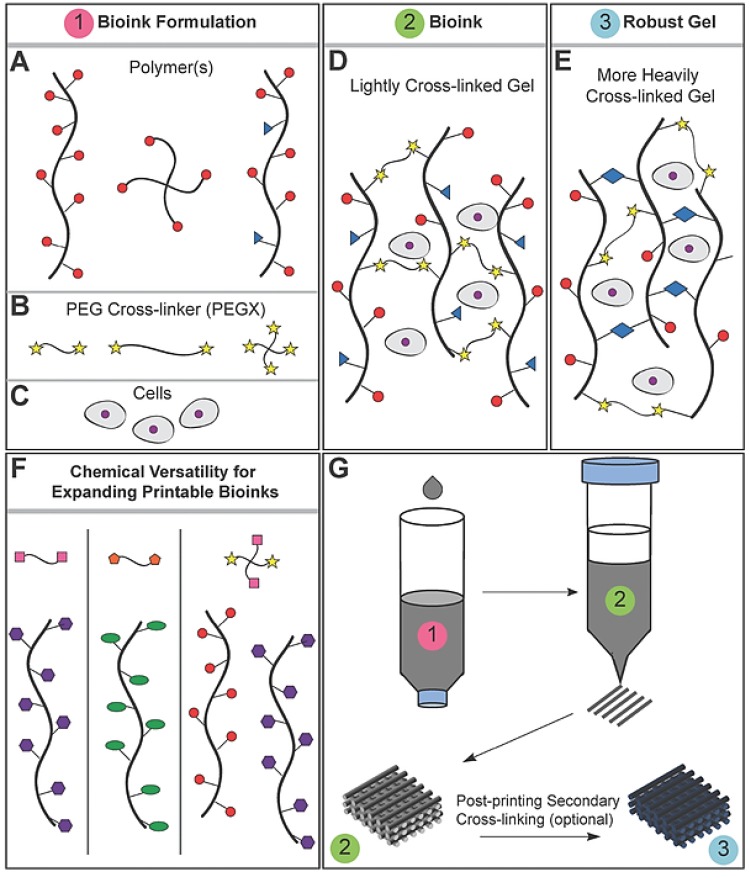
(**A**) Polymer or polymer mixtures can be linear (e.g., gelatin), branched (e.g., 4 arm PEG amine), or multifunctional (e.g., gelatin methacrylate); (**B**) PEGX can be linear or multiarm and can be various chain lengths; (**C**) Cells can be optionally incorporated by (**D**) mixing with polymers and PEGX to form the bioink; (**E**) Optional, secondary crosslinking to increase mechanical robustness may be performed postprinting; (**F**) By changing the reactive groups of PEGX, polymers of other functional groups may be crosslinked; (**G**) Printing process of PEGX bioink method and corresponding phase: PEGX with or without cells are mixed within the polymer solution and loaded into the printing cartridge. After gel formation and stable mechanical properties are achieved, gels can be 3D printed into multilayer structures. Pink: Bioink formulation, Green: Bioink, Blue: Robust gel formed after crosslinking. Reproduced with permission from [33]. Copyright 2016, John Wiley and Sons.

The major advantages of employing PEG for bioink development are hydrophilicity (supports diffusion of nutrients and exchange of gases), biocompatibility, non-immunogenicity and tailorable mechanical properties. PEG-diol has a basic structure with two hydroxyl groups which can be easily tailored into other functional groups like acrylate, azide, thiol, carboxyl and amine for wide range of applications [88]. This makes PEG versatile and it can be tailored to form a crosslinked system with PEG as the main backbone [66] or even using PEG as a crosslinker for other systems, as shown in Figure 5 [89].

Due to its low viscosity, pure PEG is difficult to be use for microextrusion bioprinting [90]. However, PEG derivatives are often integrated into other bioinks, especially as crosslinkers to enhance the mechanical strength of bioinks and to improve their bioprinting resolution. In one reported study, PEG tetraacrylates (star PEG polymer) could improve the cell laden bioprinted construct mechanical integrity more effectively (by as much as 7–8 fold) than linear polyethylene glycol diacrylate (PEGDA) [79].

Although synthetic PEG bioinks have many advantages, they lack cell binding domains. Due to this limitation, PEG is usually modified with RGD peptides to enhance cell adhesion, but these adducts have not been employed for bioprinting [91].

### 3.2. Pluronic Acid-Based Bioinks

Pluronic acid is a triblock copolymer of hydrophobic poly(propylene oxide) (PPO) segment and two hydrophilic poly(ethylene oxide) (PEO) segments arranged in a PEO-PPO-PEO configuration. It has been employed as a reverse thermosensitive hydrogel which form a solid gel at room temperature.

#### Printability and Challenges

Pluronic is a hydrophilic polymer similar to PEG. It forms a viscous hydrogel and undergoes gelation at room temperature and is only printable at high concentrations of more than 25% *w*/*v* [92]. It has been used for bioprinting applications as well as for vascularization in 3D printed constructs. The advantage of using pluronic bioinks for 3D bioprinting is the high resolution of the printed construct. Strut sizes of 150 µm [93] and 450 µm–500 µm [84] have been reported for nozzle diameters of 210 µm and 450 µm, respectively.

One drawback of pluronic acid hydrogels for bioprinting is their weak mechanical integrity, which results in the collapse of 3D printed construct. To overcome this, Dhert and coworkers have used photo-crosslinked pluronic acid to enhance the mechanical integrity of constructs [93]. Crosslinking of pluronic has been reported would also reduce the toxicity of pluronic by preventing internalization of pluronic through cell membrane [93].

Another limitation of pluronic-based bioinks is poor cell support and viability. A study reported bioprinting of bone marrow stromal cells laden pluronic bioinks with more than 90% cell death on the 3rd day [94]. Strategies to improve cell viability involve diacrylation of pluronic acid and loading of bioactive molecules into the pluronic acid. In the same study , the photo-crosslinking pluronic acid has been demonstrated to improve the viability of goat multipotent stromal cells to 31% and further improve it to 52% after loading of hydrocortisone in pluronic bioink [93]. However, the role of hydrocortisone in increasing cell viability was undetermined. Furthermore, the pluronic bioink was not able to safeguard cells from bioprinting stress, as only 60% of the human primary fibroblast survived after bioprinting [84].

In summary, pluronic bioinks face many challenges. Firstly, pluronic acid lack cell binding affinity which leads to poor cell viability. Lastly, due to poor mechanical strength, the construct fails to safeguard the cells during bioprinting and also lack shape fidelity.

### 3.3. Synthetic Nanostructured Bioinks

Nanostructured bioinks, are defined as bioinks that have at least one nanodimensional components in.

#### Printability and Challenges

Many nanostructured bioinks have been developed for bioprinting. The motivation behind introducing nanostructures is to enhance the printability, bioactivity and diffusivity of bioinks. A study has reported the blending of nanocellulose fibres with alginate bioink to result in enhanced resolution. It was observed that the viscosity was increased and the strut size decreased from 1100 µm to 500 µm. The enhanced shape fidelity was reported to be due to the increase in the storage modulus of the nanocellulose-alginate bioink [38].

Another strategy to improve the cell function involves introduction of nanosized bioactive materials. A study by Wang and coworkers reported incorporation of nanostructured hydroxyapatite in alginate-gelatin bioink to enhance the functional differentiation of human adipose derived stem cells (hADSC) into osteocytes. A significant difference has been reported in osteogenic markers in nanohydroxyapatite assisted bioinks in comparison to alginate gelatin bioink [26].

**Figure 6:**
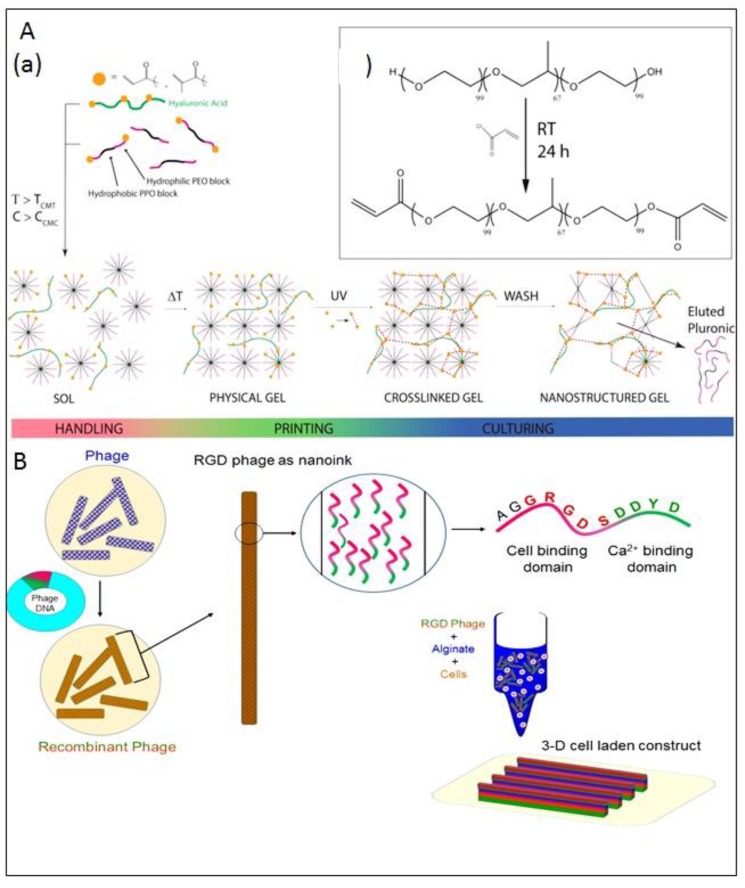
(**A**) (a) Work flow of nanostructured pluronic bioink: Pluronic is mixed with cells and other polymers such as hyaluronic acid methacrylate at a low temperature (handling regime) and is then formed to a physical gel for the printing process via temperature gelation. Subsequent UV crosslinking is then performed for mechanical stability. The crosslinked gel is washed to introduce the nanostructures and reduce the total pluronic concentration for the cell culture. (**b**) Chemical structure of pluronic F127 before and after the reaction with acryloyl chloride. Reproduced with permission from [55]. Copyright 2016, IOP Publishing; (**B**) Schematic illustration of a M13 recombinant DNA, RGD and calcium binding domains and bioink (target cells + RGD-phages + alginate) with a 3-D cell-laden scaffold printed using the phage-based bioink.

Nanostructured cavities have also been introduced into bioinks to enhance cell viability. Nanostructured cavities were introduced by introducing pluronic acrylate and pluronic F127 to hyaluronic acid methacrylate for bioprinting of chondrocytes. For nanoporous structure, pluronic was removed from the cell laden bioprinted construct by washing as shown in Figure 6A. The cell viability of this nanostructured bioink was estimated to be 88% on day 7, in comparison to pluronic methacrylate bioinks with 56.25% cell viability [55]. The nanoporous structures may have resulted in improved permeability and diffusivity, which would have increased the cell viability.

Nanostructured hybrid bioinks has also been developed for bioprinting. The blending of M13 phages with alginate to develop hybrid bioink has been successfully accomplished for bioprinting of pre-osteoblast laden constructs [95]. The M13 phages coat proteins were tailored to have integrin binding and calcium ions binding domains for better cell adhesion and alginate crosslinking. There is wide scope for the development of nanostructure bioinks and effect of nanostructure effects on cell function need to be established.

## 4. Self-Assembled Bioinks

Self-assembled bioinks are bioinks in which assembly is driven by non-covalent interaction either by its own ability or by modifying bioink with the self-assembling moieties. The efforts to mimic natural bioinks generally gets compromised by incorporation of covalent linkages to improve the printability of bioinks. Although covalent crosslinking of bioinks after or during bioprinting aid to improve shape fidelity, they often fail to mimic the natural ECM as the non-covalent interaction plays a major role in maintaining the integrity of the tissue *in vivo*. Therefore, bioinks with non-covalent interactions provide a better microenvironment for diffusion of nutrients with self-healing properties which offers a homogeneous environment. Many supramolecular assembled hydrogels have been designed for drug delivery applications, but only a few biomaterials have been employed for 3D bioprinting of cell laden constructs.

**Figure 7:**
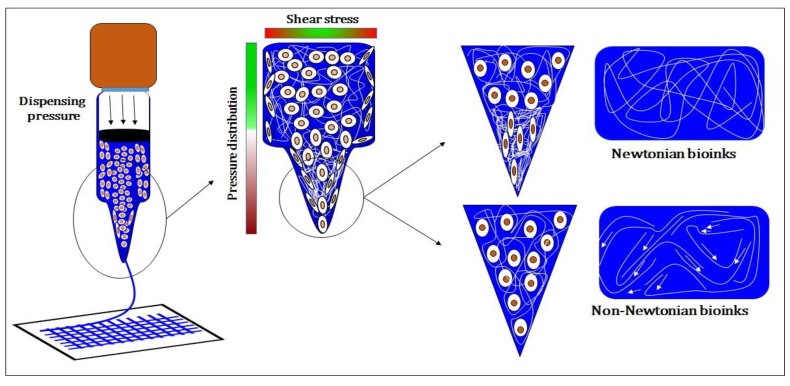
Effect of shear stress on Newtonian and non-Newtonian bioinks and distribution of shear stress and pressure distribution inside syringe.

Cell viability, shape fidelity and resolution are important parameters which dictate the printability of bioinks. Photopolymerization of bioinks such as GELMA, HAMA and PEGMA by UV/visible light exposure after bioprinting seems to be one of the best methods to achieve improved shape fidelity, but on the other hand cell viability gets compromised due to the presence of photo-initiator [73], crosslinks and UV exposure. To overcome this, hydrogels were designed to avoid UV exposure as well as prevent photo-initiator usage. Self-assembled hydrogels that have been designed using self-assembled moieties like cucurbiturils, cyclodextrins, DNA *etc.* represent a suitable alternative to overcome this problem. Earlier they were designed as injectable hydrogels, but later researchers realized that similar hydrogels could be exploited for 3D bioprinting. For injectable hydrogels, the shear thinning properties of hydrogels among the essential properties, like for bioinks. Shear thinning of bioinks is a solution to blockage of nozzles during bioprinting. It also decreases the effect of shear stress on the encapsulated cells, as shown in Figure 7. Due to its shear thinning properties, the viscosity of the bioink decreases, which can safeguard the cells from shear stress that is present majorly near the walls of cartridge and nozzle. Another hazard to cells is due to the pressure drop when the bioink enters the syringe from the nozzle. Effect of this pressure drop get normalized due to sliding of bioinks chains at the junction (*i.e.*, event of shear thinning) and also enhances the resolution by providing a smooth flow through the nozzle.

### Printability and Challenges

**Figure 8:**
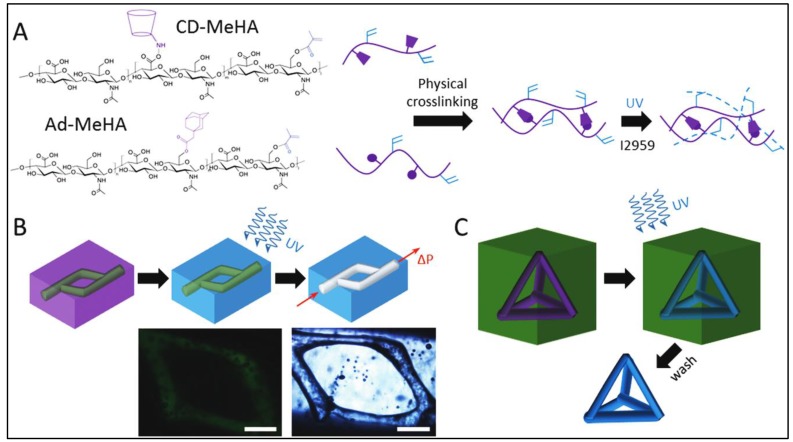
(**A**) Hyaluronic acid modified with both methacrylates (blue) and guest-host molecules (purple). Ad–MeHA and CD–MeHA macromers crosslink by both physical bonding upon mixing and through a secondary crosslinking of methacrylates with UV light exposure. (**B**) The printing of channels, by writing an ink into a support gel that is modified for secondary crosslinking. UV irradiation covalently crosslinks the support gel around the ink. Pressure driven flow results in removal of the ink, leaving a channel network (**C**) The printing of self-supporting structures, by writing an ink that can be covalently crosslinked into a support gel, followed by UV crosslinking, and dissolution of the support with excess β-CD. Scalebars: 500 μm. Reproduced with permission from [22]. Copyright 2016, John Wiley and Sons.

For self-assembled bioink development, natural polymers or peptides have been modified with self-assembled host guest moieties like cyclodextrin-adamantane, cucurbiturils-diaminohexane, *etc.* Self-assembly moieties have strong non-covalent interactions between them that help improve the viscosity of bioinks. The advantages of self-assembled bioinks include shear thinning, self-healing properties, good cell safe-guarding, homogeneity and temperature sensitivity, although, a common challenge in using self-assembled bioinks in the low bioprinting resolution. For instance, a study reported a large strut size of 500 µm for DNA-peptide-based bioink [40]. Attempts at high resolution bioprinting of self-assembled bioinks involve the use of the host guest chemistry of cyclodextrin-adamantane. In a particular study conducted by Burdick and workers, the use of cyclodextrin-adamantane (40%) modified hyaluronic acid (HA)-based supporting gel for bioprinting of cell laden cyclodextrin-adamantane (25%) modified HA methacrylamide hydrogel shown in Figure 8 was demonstrated. For this study, at 38.9 ± 7 nL/mm bioprinting speed, strut diameter of 222.6 ± 29.7 µm have been printed using a 27 gauge nozzle (410 µm), demonstrating high resolution [22].

Another disadvantage of self-assembled bioinks is low shape fidelity. One efficient approach involves modification of hydrogel with methacrylamide group for photo-crosslinking along with self-assembled moieties. In the same abovementioned study, cyclodextrin (CD)-adamantane (Ad) functionalized hyaluronic acid (HA) was modified with methacrylamide (Me) to form Ad–MeHA and CD-MeHA for covalent crosslinking by UV exposure and used to form fluidic channels as well as 3D printed constructs [22]. 3D printed construct was printed using methacrylamide modified Cd-Ad hyaluronic acid bioink for printing in supporting gel, which can be removed after crosslinking. The application of cucurbiturils-diaminohexane modified hyaluronic acid has also been demonstrated to be used as bioprinted scaffolds for better shape fidelity and stability of cell laden constructs [39].

Self-assembled bioinks have good cell safe-guarding potential from stress during bioprinting. The bioprinting of pluripotent stem cells laden DNA-polypeptide bioink achieved >98% viability when bioprinted with valve-based nozzles [40]. Another study reported >90% viability of human fibroblasts bioprinted with Cd-Ad-modified hyaluronic acid, demonstrating the cell guarding ability ofself-assembled bioinks [22]. Self-assembled bioinks have enormous potential for bioprinting applications. However, as only few studies have been reported for bioprinting of cell laden constructs, there is a need to further explore and optimize self-assembled bioinks.

## 5. Summary and Conclusions

Bioink development has enormous scope for advancement and application. However, many challenges remain ahead. For every bioink developed, a new set of printing parameters has to be optimized individually for a type of cell line. Each cell type needs a unique set of bioprinting parameters and bioink properties. Although there are several reports which have tried to model the optimization of bioinks, a generalized model cannot fulfill the requirement of all cell types. For the development of cell-specific bioinks, studying the cells’ mechanical characteristics under printing stress and their fate will help in developing a better understanding for further improvements. Some studies have reported the characteristics of various cell types thorough atomic force microscopy and have shown significant diversity in mechanical characteristics of different cell types [19,20,96,97,98]. Moreover, bioinks with improved cell shielding properties are still largely unexplored. Studies have clearly demonstrated the negative effects of printing stress on cell growth and function [2,9,41,42,43]. Development of bioinks with the right balance of rheological properties for stress shielding and printability has to be established.

Besides this, advanced functional cues need to be developed for concurrent sensing of cell function with the growth pattern in the bioprinted tissue or organ construct. Monitoring of cell growth and function with time will help in developing a better understanding of intercellular and intracellular phenomenon and efficacy of bioinks in supporting such interactions. This will open an avenue to move a step further towards development of functional organs and tissues for implantation. Perhaps current technologies that have been developed to monitor activity of cells as a function of time like organ on a chip [36,62] which have continuous perfusion system helping in monitoring of the system could be adapted for 3D bioprinted tissue constructs. Integration of smart systems with the bioinks without compromising the cell function and growth may fulfill this objective. Also, the development of smart materials along with systems would further contribute in stepping further towards bioprinting of functional tissue and organs. A few smart materials for 4D printing have been developed which change their properties and shape in response to external stimuli [99]. Similar change in property phenomenon with respect to internal stimuli produces by the cells would bolster in providing a cell supportive environment.

Furthermore, scaling up to print large structures, e.g., organs for transplantation, needs further development. There are many challenges in such structures such as sufficient mechanical integrity, time constraints due to cell viability, mechanism(s) of nutrient diffusion such as vascularisations and many more. The idea of organ bioprinting not only needs innovative design and methods but also suitable bioinks. Parallel development in all the areas should be able to turn the idea into reality.

In conclusion, bioprinting 3D tissue and organ structures can only be accomplished by overcoming the present challenges in printability and by understanding the cellular bioprocesses which takes place in response to a given bioink/ECM. The foremost challenge is the printability of bioinks because it is the first step towards fabrication of complex structures. Natural bioink printability needs to be optimized for different cell lines and relationships to be established between the bioinks’ microstructure and cell structural integrity with their function. Bioinks with better printability and diffusivity have to be developed for the development of large structures and efficient nutrient delivery. The relations between bioprinting parameters and bioink properties need to be established to determine condition for making constructs with better shape fidelity and resolution.

## Figures and Tables

**Table 1:** Different strategies to improve the printability of bioinks.

SNo.	Motivation	Strategies	Advantages	Disadvantages	Reference
**1**	To improve bioink printability	Blending	Improve the viscosity of bioink	Decreases cell viability	[23,24,25,26,27,28,29]
		Interpenetrating hydrogels	Improve viscosity of bioink	Decreases cell viability	[30,31]
		Partial crosslinking by synthetic polymers	Improve viscosity, resolution	Involves synthetic cross-linkers which limits cell viability and have lower overall mechanical properties	[32,33]
		Co-axial nozzle	Printing of low viscosity bioinks, improve resolution, facilitate heterogeneous construct fabrication, enable multimaterial deposition	Low cell viability during bioprinting	[34]
		Cell loaded micro-carriers laden bioink	Low viscosity bioinks can be printed, improve mechanical strength of bioink, good cell viability and function, enable heterogeneous construct fabrication	Limited resolution	[35]
		Cell spheroids laden bioink	Efficient control over dimensions of printed construct, reduce shear stress during printing and consistent diffusion of bioactive molecules and good cell viability	Large constructs cannot be fabricated, comparatively large structures which limits bioprinting resolution, unable to form multilayered structures	[36,37]
		Nanoparticles loaded bioinks	Improve viscosity with shear thinning properties, better resolution, safeguarding of cells from printing stress and cell function	Low cell viability after bioprinting	[26,38]
**2**	To improve shape fidelity	Modification with Self assembled moieties	Improve safe-guarding of cells from printing stresses	Difficult to control properties of self-assembled bioink	[22,39,40]
		Optimization to quantify shear stress during bioprinting	Improve cell viability during bioprinting and determine printing parameters for better resolution	Optimization was established for limited bioinks and are not applicable for other bioinks	[2,9,23,24,41,42,43,44,45]
		Photo-chemical Crosslinking	Increased mechanical strength to hold printed 3D structure	Decrease cell viability	[29,46,47,48]
		Ionic crosslinking	Increase mechanical strength	Inefficient for multilayered constructs, decreases cell viability	[25,43,45,49,50]
		Concomitantly printed scaffolds	Improve mechanical strength and efficient diffusivity of construct	Involve synthetic bioinks	[48,51]
		Sacrificial scaffolds	Facilitate printing of complex shapes, efficient diffusion characteristics	Toxicity in case of synthetic scaffolds	[49,52,53,54]
		Bioink for nano-porous scaffolds	Improve diffusion of nutrients and shape fidelity	Toxic synthetic polymers	[55]
		Crosslinking with supportive scaffold	Improve mechanical strength and stability, implantable constructs due to efficient degradation	Rapid degradation	[56]
**3**	To achieve good cell viability and function	RGD modification	Improve cell adhesion and viability	-	[57,58]
		Blending with biopolymers with cell binding domains	Improve cell binding as well as viability	Decrease printability	[33,48]
		Decellularized ECM	Provide native microenvironment to laden cells	Low viscosity and poor resolution	[59,60]
		Printing temperature	Improve cell viability during bioprinting	Decrease the printability of bioink	[23]
		Vascularized constructs	Better diffusion characteristics which improves the cell viability	-	[53,55]

**Table 2:** Bioinks for micro-extrusion bioprinting.

Source	Bioinks	Crosslinking Mechanism(s)	Shape Fidelity	Printing Resolution	Cell Supportive Ability	Drawbacks	Reference
Nature derived bioink	Alginate	Ionic	Medium	400–600 μm (by blending)	Low	Lack cell binding domains	[31]
	Gelatin	Covalent, enzymatic, physical and thermal	Medium	350–450 μm (partial crosslinking and blending)	High	Low viscosity and mechanical integrity	[44,50,54,56,61,62]
	Collagen I	Physical	Low	1000 μm	High	Low viscosity and mechanical integrity	[21]
	Fibrin	Enzyme based	High	-	Medium	Fast biodegradation and irreversible gelation	[48,58]
	Hyaluronic acid	Physical or covalent	High	730 ± 28 μm (Semi-IPN)	High	Low stability	[50,63]
	Decellularied extracellular matrix	Physical	Low	-	High	Low viscosity and mechanical strength	[59]
	Silk	Enzymatic or physical	High	280–320 μm	Low	Lack cell binding domains, Low cell viability	[64,65]
	Chitosan	Ionic	low	400 μm (blending)	Medium	Low mechanical integrity, low cell viability	[60,66]
Synthetic bioink	PEG	Covalent	-	-	Medium	Lack cell binding domains	[44]
	Pluronic acid	Covalent	High	150 μm	Low	Lack cell binding domains, Low cell viability and mechanical strength	[67]
	Nanostructured bioinks	Physical	Medium	-	Medium	-	[68,69]
Self-Assembled bioink	Cd-Ad HA	Non-covalent	Medium	222.6 ± 29.7 μm (supporting gel)	Medium	Low mechanical strength	[28]
	DNA-peptide	Non-covalent	Medium	500 μm	Medium	Low mechanical integrity and viscosity	[70]
